# *Colpomenia sinuosa* extract mitigates lead acetate-induced testicular dysfunctions in male rats

**DOI:** 10.3389/fmolb.2025.1551773

**Published:** 2025-04-28

**Authors:** Layla A. Almutairi, Amal S. Abu-Almakarem, Noorah Saleh Al-Sowayan, Sahar Abdulrahman Alkhodair, Hayat M. Albishi, Thamir M. Eid, Fahad A. Alshanbari, Najlaa Yousef Abuzinadah, Maysa A. Mobasher, Karim Samy El-Said

**Affiliations:** ^1^ Department of Biology, College of Science, Princess Nourah bint Abdulrahman University, Riyadh, Saudi Arabia; ^2^ Department of Basic Medical Sciences, Faculty of Applied Medical Sciences, Al-Baha University, Al-Baha, Saudi Arabia; ^3^ Department of Biology, College of Science, Qassim University, Buraidah, Saudi Arabia; ^4^ Department of Biochemistry, Faculty of Science, King Abdulaziz University, Jeddah, Saudi Arabia; ^5^ Department of Medical Biosciences, College of Veterinary Medicine, Qassim University, Buraydah, Saudi Arabia; ^6^ Department of Biological Science, College of Science, University of Jeddah, Jeddah, Saudi Arabia; ^7^ Department of Pathology, Biochemistry Division, College of Medicine, Jouf University, Sakaka, Saudi Arabia; ^8^ Biochemistry Division, Chemistry Department, Faculty of Science, Tanta University, Tanta, Egypt

**Keywords:** *Colpomenia sinuosa*, Brown algae, antioxidant, anti-inflammatory, lead, testicular dysfunctions

## Abstract

**Background:**

*Colpomenia sinuosa* brown alga contains pharmacologically active compounds with a wide spectrum of bioactivities; however, few studies have been conducted in the Mediterranean to assess their effects against heavy metal toxicity. One common non-biodegradable contaminant that poses a serious risk to human health and the environment is lead (Pb). This study investigated the efficacy of *C. sinuosa* extract (CSE) treatment on testicular injury caused by lead acetate (PbAc) in rats.

**Methods:**

The phytochemical, GC/MS profiling, and metal chelation ability of CSE were evaluated. Molecular docking studies were performed using AutoDock Vina. The oral LD_50_ of CSE was determined by probit analysis. 40 male rats were used as follows: Gp1 as a negative control; Gp2 was treated with 1/10 of CSE LD_50_ (340 mg/kg b. wt.); Gp3 was administered PbAc solution (100 mg/kg b. wt.); Gp4 was orally administered PbAc as in Gp3 and CSE as in Gp2. All treatments were given daily by gastric tube for 30 days. Body weight changes, biochemical, molecular, and histopathological examinations were investigated.

**Results:**

The results demonstrate that CSE exerted a pronounced metal-chelating activity *in vitro* and contain promising phytochemicals. The LD_50_ of CSE was 3,400 mg/kg b. wt. PbAc-treated rats reported significant testicular dysfunction with impaired semen analysis, biochemical, molecular, and histological changes. CSE treatment showed significant palliative effects on these dysfunctions via improvements in antioxidant status, anti-inflammatory properties, and histopathological alterations. Interestingly, CSE treatment modulates the JAK2/STAT3, and NLRP3/Caspase-1 pathways axis in PbAc-injured rats.

**Conclusion:**

This study for the first time investigated the biochemical and molecular mechanisms regarding the effects of CSE treatment on PbAc-induced testicular damage in male rats. CSE showed potential attenuative effect on the testis injury induced by PbAc treatment by targeting JAK2/STAT3, and NLRP3/Caspase-1 pathways. These findings suggest that CSE could be used against the adverse effect of PbAc on male repro-toxicity.

## 1 Introduction

Heavy metal contamination has emerged as a major global environmental concern due to industrialization, threatening animals and humans (Das et al., 2023). Various risks of lead (Pb) exposure exist including painting, battery repair, metal smelting, glass blowing, welding, pesticides, medicines, and cosmetics. The WHO remains concerned about exposure to Pb in contaminated environments (Kumar and Prasad, 2018). Exposure to Pb accounts for 0.6% of the global burden of illness, resulting in 540,000 deaths worldwide in 2016 (Nag and Cummins, 2022). Male infertility is a major concern with significant clinical problems affecting approximately 50–80 million people worldwide, with male factors accounting for roughly 20%–30% of all instances (Babakhanzadeh et al., 2020). Pb tends to accumulate in the tissues; semen alterations can be induced in Pb-exposed workers through oxidative stress (Giulioni et al., 2023). Furthermore, Pb is an endocrine disruptor negatively affecting endocrine functions, sperm production, and fertility. Some studies have correlated Pb exposure with male infertility (Lin et al., 2023; Mobasher et al., 2024). The accumulation of Pb in Sertoli cells alters glycolysis and lactate transport, causing reproductive toxicity (Yu et al., 2019). Pb-induced oxidative stress has been linked to male reproductive dysfunctions, which in turn, results in a testicular antioxidant deficit in rats. A previous study reported testicular tissue atrophy in rats exposed to lead acetate (PbAc), which disrupts spermatozoa maturation through oxidative stress (Abu-Khudir et al., 2023). It has been demonstrated that PbAc exposure caused male reproductive damage by increasing inflammatory mediators (Akarsu et al., 2023).

Natural constituents have been used in recent years, and various lines of research have proved their usefulness in dampening Pb-induced testicular damage (Daud et al., 2020; Abu-Khudir et al., 2023; Mobasher et al., 2024). Marine-derived natural products are rich sources of secondary metabolites that have shown pronounced novel therapeutic agents (Zhong et al., 2021). Marine algae have been used as novel biological materials and have attracted a great deal of interest in biomedical applications due to their contents of biologically active compounds (Wang et al., 2017). These compounds have been reported to exert several pharmaceutical potentials, including antioxidant, antitumor, antidiabetic, anti-inflammatory, and antimicrobial properties (Carpena et al., 2022). Atya et al. (2021) demonstrated that *Colpomenia sinuosa* had potential antitumor properties on hepatic cancer cells, significantly improving liver functions, hepatic inflammation, and oxidative stress. Additionally, it has been reported that *C. sinuosa* is a source of bioactive compounds possessing apoptotic, anti-migratory, and antibacterial efficacy (Al Monla et al., 2020). Lekameera et al. (2012) suggested the antioxidant potential of *C. sinuosa* extract and its nephroprotective effect in rats. A previous study reported that the red algae extract improved testicular functions by down-regulating caspase-3 gene expression and inducing antioxidant enzymes in rats (Renda Sari et al., 2023).

The JAK/STAT pathway influences male reproductive processes; JAK2/STAT3 activation has been reported to inhibit the spermatogenic process. In contrast, JAK2 inhibition decreased oxidative stress, apoptosis, and improved spermatogenesis (Alnajem and Al-Maghrebi, 2023). A prior study revealed the anti-inflammatory benefits of natural constituents against testicular inflammation by decreasing JAK-STAT signaling (Somade et al., 2023; Mobasher et al., 2024). Moreover, it has been documented that arbutin alleviated PbAc-promoted testicular injury by targeting the JAK2/STAT3 pathway in rats. Pro-inflammatory inflammasomes have been implicated in the progression of testicular injury by Pb exposure, with NLRP3 being the most common type, including an effector protein called caspase-1 (Arab et al., 2024). A study concluded that diacerein significantly protected testicular tissues of rats exposed to cadmium by modulating NLRP3/caspase-1 pathways of inflammation and apoptosis (Fouad et al., 2020). Interestingly, CSE showed various bio-medical potentials. However, the roles of CSE in ameliorating Pb-triggered testicular toxicity have not been previously reported. Therefore, this novel study addressed the efficacy of CSE for the first time on mitigating testicular injury promoted in rats by PbAc through targeting oxidative stress, inflammation, JAK2/STAT3, and NLRP3/Caspase-1 pathways.

## 2 Materials and methods

### 2.1 Chemicals

Lead acetate (PbAc) (≥99.0% trace metals basis, Cat. no. 6080-56-4) was purchased from Sigma-Aldrich (St. Louis, Missouri, United States). Aluminum chloride, potassium acetate, phenol, and sodium nitroprusside were purchased from Merck Company (Darmstadt, Germany).

### 2.2 Collection of *C. sinuosa* and extract preparation

*C sinuosa alga* were collected from the Egyptian Mediterranean coast of Alexandria, at Abu Qir Bay, and the Eastern Harbor during the winter of 2023. Samples were transported in an icebox to the laboratory at the Faculty of Science, Tanta University in Tanta City, Egypt. The identification of *C. sinuosa* was conducted following Aleem (1993) and confirmed using the Algae Base website (Guiry and Guiry, 2021). After the sample collection, they were washed with distilled water, dried in the shade, ground into a fine powder, and stored for analysis. A 50 g powdered sample was soaked in 500 mL of 70% ethanol at room temperature for 1 week with continuous shaking. The extracted materials were then filtered and evaporated under reduced pressure at 45 °C to obtain the *C. sinuosa* extract (CSE), which was stored at 4°C for subsequent studies (Hassan and Shobier, 2018).

### 2.3 Phytochemical analysis of CSE

The metal chelation ability of CSE was determined, the extract binds with ferrous ions (Fe^2+^) generated *in vitro* using iron sulfate as ions donor, then Fe^2+^ ions form a deep red solution with 1,10-phenanthroline. The absorbance at 510 nm spectrophotometrically against control. The % of chelation activity = A_C_ – A_S_/A_C_ ×100. A_C_: Absorbance of control, A_S_: Absorbance of CSE (Moukette et al., 2015). The total phenol, flavonoid, total antioxidant capacity, saponin, and anthocyanin were determined (Hiai et al., 1975; Singleton et al., 1999; Prieto et al., 1999; Ebrahimzadeh et al., 2008). Additionally, spectrophotometric analysis was used to assess its DPPH radical scavenging capabilities (Blois, 1958; El-Said et al., 2023).

### 2.4 Gas chromatography and mass spectrum (GC-MS) profiling of CSE

Using the Trace GC 1310-ISQ mass spectrometer, (Thermo Scientific, Austin, TX, United States), with a direct capillary column TG-5MS (30 m × 0.25 mm × 1.0 µm thickness), the phytochemicals present in CSE were identified by comparing their mass spectra and retention periods to those in WILEY 09 and NIST 11. The injector and MS transfer line temperatures were kept at 270, 260°C, respectively. Helium was used as a carrier gas at a constant flow rate of 1 m/min. The solvent delay was 3 min and diluted samples of 1 µL were injected automatically using Auto sampler AS1300 coupled with GC in the split mode. EL mass spectra were collected at 70 eV ionization voltages. Mass spectrum was scanned in the m/z range of 30–350 amu in full scan mode (Li et al., 2021).

### 2.5 Determination of the median lethal dose (LD_50_) of CSE

Twenty male Sprague Dawley rats (160 ± 10 g) were separated into 5 groups (n = 4). The LD_50_ of CSE after oral treatment was determined. These groups received CSE injections ranging from 1,000 to 5,000 mg/kg body weight. The experiment was conducted in full compliance with international and institutional ethical guidelines and regulatory standards according to OECD 423 (acute oral toxicity–acute toxic class method) guidelines. The use of the LD_50_ test was necessary due to necessity for risk assessment. We minimized animal suffering by implementing humane endpoints, continuous monitoring, and following refinement strategies to reduce distress. Rats were then monitored for 24 h to monitor signs of poisoning, including behavior, physical appearance, lethargy, tremors, convulsions, or death. The probit analysis determined the LD_50_ value (Finney and Stevens, 1948).

### 2.6 Animals and experimental design

Forty mature male Sprague Dawley rats (160 ± 10 g, 6 weeks age) were purchased from the National Research Center in Cairo, Egypt. This investigation was carried out in accordance with the Faculty of Science’s Research Ethical Committee rules (IACUC-SCI-TU-0264). The rats were divided into four equal groups, with the 1^st^ group (Gp1) serving as a negative control group. Gp2 was orally gavaged with CSE dissolved in distilled water (340 mg/kg body weight) (1/10 of LD_50_). Gp3 was orally administered with a solution of PbAc (100 mg/kg body weight) (Ayuba et al., 2017). Gp4 was treated with CSE as in Gp2 24 h after the last administration of PbAc (100 mg/kg body weight). At the end of the experiment, after a month, the percentages of body weight (% b. wt.) changes were recorded. Rats were slaughtered under isoflurane (3%–5%) anesthesia in oxygen by inhalation in a closed chamber then the concentration was reduced to a lower level, around 1%–2% isoflurane in oxygen, to maintain anesthesia during the procedure.

#### 2.6.1 Sampling

Blood samples were collected by cardiac puncture, centrifuged at 4°C to separate serum samples, which were then stored at −20°C for subsequent assays. The testicular and epididymal tissues were carefully excised, washed in phosphate-buffered saline (PBS), and their weights were recorded, stored at −80°C for future analysis. Sperm abnormalities, count, viability, and Carrageenan motility were evaluated. Parts of rat testicular tissue were used for molecular and biochemical investigation and other parts were preserved in 10% buffered formalin for histological analysisThe testicular tissues were homogenized in cold PBS containing protease inhibitors (aprotinin), using a homogenizer. The homogenate (10%) was centrifuged at 10,000 × g for 10 min at 4°C, the resulting supernatant was carefully collected and transferred to a clean tube, avoiding any pellet contamination, then stored at −80°C. The serum Pb concentrations and Pb residues in testicular homogenate were measured after various treatments using a PerkinElmer 2380 atomic absorption spectrophotometer.

### 2.7 Evaluation of spermatogenic parameters

Sperm samples were stained with Wells and Awa’s stain to evaluate abnormalities. The diluted epididymal sperm suspension (1:5 dilution in a sterile, pre-warmed phosphate buffer saline, (37°C)) to a final volume (1 mL) was gently mixed to ensure distribution of sperm cells, then 10 µL aliquot of the sperm suspension was placed on a hemocytometer, examined under a light microscope (×400 magnification) to assess sperm motility. Sperm’s vitality was determined using the eosin stain by dye exclusion test (Wells and Awam, 1970).

### 2.8 Biochemical analysis

Luteinizing hormone (LH) (Cat. no. E-EL-R0026), Follicular stimulating hormone (FSH) (Cat. no. MBS2021901), and testosterone levels (Cat. no. 80550) were determined in serum using their ELISA kits from Elabscience (Houston, Texas, 77,079, United States). The levels of malondialdehyde (MDA) (Cat. no. MD2529), protein carbonyl (PC) (Cat. no. MBS2600784), superoxide dismutase (SOD) (Cat. no. SD2521), and catalase (CAT) (Cat. no. CA2517) were determined in the testicular supernatant Biodiagnostic (Cairo, Egypt). Rats’ ELISA kits from MyBioSource, Inc., San Diego, CA, United States, were used for measuring steroidogenic acute regulatory protein (StAR) (Cat. no. MBS729454), 3β-hydroxysteroid dehydrogenase (3β-HSD) (Cat. no. MBS2604899), and 17β-HSD (Cat. no. MBS2104946) sorbitol dehydrogenase (SDH) (Cat. no. MBS269299), lactate dehydrogenase (LDH) (Cat. no. MBS269777), glutathione (GSH) (Cat. no. MBS265966), glutathione peroxidase (GPX) (Cat. no. MBS744364), and glutathione-S-transferase (Cat. no. MBS8243172). Furthermore, rats’ ELISA kits were used to determine inflammatory biomarkers in the testicular homogenate, including interleukin-6 (IL-6) (Cat. no. E-HSEL-R0004), tumor necrosis factor-α (TNF-α) (Cat. no. RAB0479), nuclear factor kappa-B (NF-κB) (Cat. no. MBS453975), and cyclooxygenase-2 (COX-2) (Cat. no. MBS266603). The levels of NLRP3 (Cat. no. MBS2033695), cleaved-caspase-1 (Cat. no. MBS7255117), and Phospho-JAK2 (Cat. no. MBS7269637) were determined by using their rat’s ELISA kit from MyBioSource, Inc., San Diego, CA, United States. Phospho-STAT3 (Cat. no. RAB0446) ELISA kit was used for evaluating phosphorylation of STAT3, purchased from Sigma-Aldrich (Chemie GmbH Eschenstrasse 5 D-82024, Taufkirchen, Deutschland).

### 2.9 Molecular analysis

The testicular tissues were cut under sterile conditions, snap-frozen in liquid nitrogen to prevent genetic materials degradation, immediately placed in sterile RNase-free PBS, stored at −80°C. The mRNA expressions of JAK2, STAT3, NLRP3, Caspase-1, Bcl-2, and Bax genes were evaluated in testicular tissues using SYBR Green. Pure RNA was extracted using a total RNA purification kit (Thermo Scientific, Fermentas #K0731). Total RNA is routinely used in cDNA synthesis, and then their concentrations were quantified using nanodrops. The isolated cDNA was amplified using 2X Maxima SYBR Green/ROX qPCR Master Mix following the manufacturer protocol (Thermo Scientific, United States #K0221). Primers were prepared using the Primer-Blast program from NCBI with β-actin gene as housekeeping gene (Table 1). The Light Cycler 480 System (Roche, Indianapolis, IN) was used under the following cycling conditions: 1 cycle at 95°C for 5 min and then 45 cycles at 95°C for 10 s, 60°C for 30 s, and 72°C for 10 s. The relative expression of target genes was calculated using the Livak and Schmittgen (2001) method.

**TABLE 1:** The forward and reverse primer sequences for RT-PCR.

Gene	Accession no.	Forward sequence (5′–3′)	Reverse sequence (5′–3′)
*JAK2*	NM_031514	CCAGCCGTGCTTGAAAACAT	CTCAACGGCAAAGGTCAGGA
*STAT3*	NM_012747	CACCCTGAAGCTGACCCAG	TATTGCTGCAGGTCGTTGGT
*NLRP3*	NM_001191642	ACGGCAAGTTCGAAAAAGGC	AGACCTCGGCAGAAGCTAGA
*Caspase-1*	NM_012762	GACAAGATCCTGAGGGCAAA	GGTCTCGTGCCTTTTCCATA
*β-actin*	NM_031144	ATCGCTGACAGGATGCAGAAG	AGAGCCACCAATCCACACAGA

JAK2, Janus kinase-2; STAT3, Signal transducer and activator of transcription-3; NLRP3, Nucleoside-binding oligomerization domain, leucine-rich repeats and pyrin domain containing protein 3.

### 2.10 Molecular docking analysis

The structures of ligands were retrieved from PubChem database in SDF format and 3D structures of proteins were energy-minimized using Avogadro 1.2.0 software with the MMFF94 force field (Hanwell et al., 2012). The structure of the JAK2 (UniProt ID: Q62689), STAT3 (UniProt ID: P52631), NLRP3 (UniProt ID: D4A523) and CASP1 (UniProt ID: P43527) proteins were retrieved from the UniProt database. The binding sites for these proteins were predicted based on literature information and validated using the CB-DOCK2. Proteins were pre-pared for docking using AutoDock Tools 1.5.7 (Morris et al., 2009). Molecular docking studies were performed using AutoDock Vina to predict the binding modes and affinities of the compounds with each protein (Trott and Olson, 2010). The grid boxes for docking were centered on the predicted binding sites. The exhaustiveness parameter was set to 8, and the default scoring function was used for the docking calculations. The docked complexes were visualized and analyzed using BIOVIA Discovery Studio Visualizer 2020 (San Diego, CA, United States). The binding affinities (ΔG values) and intermolecular interactions, including hydrogen bonds and hydrophobic interactions, were analyzed and reported.

### 2.11 Histopathological investigations

Testis tissues were fixed in 10% buffered formalin, washed with xylene, embedded in paraffin wax, and sectioned at 5 μm. Sections were stained with hematoxylin and eosin (H&E), inspected with a light microscope (Olympus CX31, Japan), and photographed with a digital camera (Olympus Camedia 5,060, Japan) (Bancroft and Gamble, 2008). The Johnsen scoring system was used to assess the germinal epithelial cell architecture of the seminiferous tubules and the number of Sertoli cells in the testis (Johnsen, 1970).

### 2.12 Statistical analysis

A one-way analysis of variance (ANOVA) or Kruskal-Wallis tests were conducted to determine significant differences, and the results were analyzed using GraphPad Prism software (San Diego, CA, United States). Tukey’s test was performed for multiple comparisons, with P < 0.05 considered statistically significant.

## 3 Results

### 3.1 Phytochemical composition of CSE

*C. sinuosa* powder (CSP) yield an adequate amount of its extract (12%). The metal chelating activity of CSP was 85% ± 3.56, and its EC50 was 340.79 ± 4.38 μg/mL. The total antioxidant capacity (TAC) of the extract was 78.93 ± 3.78 mg AAE/g DW. The total phenolic compounds and flavonoid contents of CSP were 33.21 ± 2.43 mg GAE/g DW and 21.48 ± 1.95 mg QE/g DW, respectively. The DPPH scavenging activity was 87% ± 4.38 and its IC50 was 5.74 ± 0.73 mg/mL (Table 2).

**TABLE 2:** Quantitative phytochemical analysis of *C. sinuosa* powder (CSP).

Phytochemical analysis	CSP
Metal chelating activity (MCA) (%)	85% ± 3.56
EC_50_ of MCA (μg/mL)	340.79 ± 4.38
Total antioxidant capacity (TAC) (mg AAE/g DW)	78.93 ± 3.78
Total phenolic content (mg GAE/g DW)	33.21 ± 2.43
Total flavonoids contents (mg QE/g DW)	21.48 ± 1.95
DPPH scavenging activity (%)	87% ± 4.38
IC_50_ of DPPH (mg/mL)	5.74 ± 0.73

CSP, *Colpomenia sinuosa* powder, MCA, metal chelating activity, EC_50_, Half maximal effective concentration, GAE, gallic acid equivalent, QE, quercetin equivalents, DW, dry weight, TAC, total antioxidant capacity, ECG, epicatechin gallate, AAE, ascorbic acid equivalent, DPPH, Diphenyl-1-picrylhydrazyl, IC_50_, Half maximal inhibitory concentration.

### 3.2 Gas-chromatography mass spectrometry profiling of CSE

**FIGURE 1:**
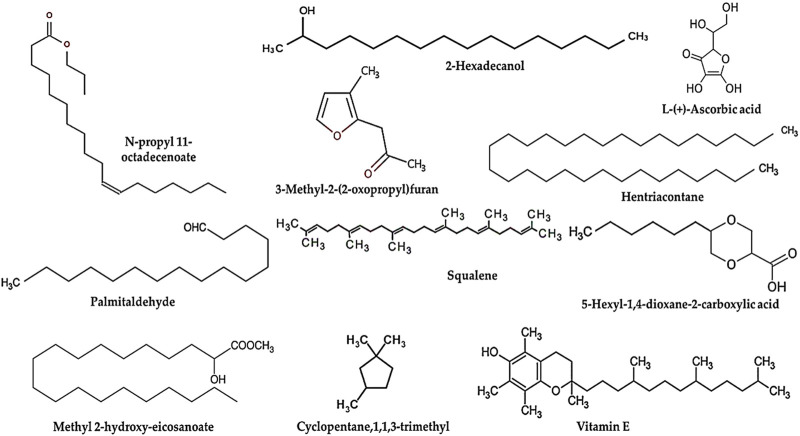
The most abundant chemical constituents in *Colpomenia sinuosa* extract (CSE), highlighting the most abundant bioactive compounds that were detected by gas-chromatography mass spectrometry analysis measured in terms of peak area.

The CSE contains pronounced chemical constituents. The abundant compounds detected in CSE were 2-Hexadecanol, L-Ascorbic acid, N-propyl 11-octadecenoate, 3-Methyl-2-(2-oxopropyl)furan, Hentriacontane, Palmitaldehyde, Squalene, 5-Hexyl-1,4-dioxane-2-carboxylic acid, Methyl 2-hydroxy-eicosanoate, Cyclopentane,1,1,3-trimethyl, and Vitamin E. Their retention time (RT) were 16.78, 17.86, 19.52, 19.94, 22.53, 22.79, 23.86, 24.52, 25.24, 25.89, and 28.21 min, respectively. The peak area percentages (PA%) were 4.69%, 24.57%, 8.91%, 3.69%, 10.59%, 5.08%, 3.27%, 5.18%, 3.47%, 2.56%, and 3.89%, respectively (Figure 1; Table 3).

**TABLE 3:** Chemicals compounds in CSE by GC-MS analysis.

No.	RT (min.)	Name	MF.	P.A (%)
1	16.78	2-Hexadecanol	C_16_H_34_O	4.69
2	17.81	L-(+)-Ascorbic acid	C_6_H_8_O_6_	24.57
3	19.52	N-propyl 11-octadecenoate	C_12_H_40_O_2_	8.91
4	19.94	3-Methyl-2-(2-oxopropyl)furan	C_8_H_10_O_2_	3.69
5	21.64	Hexadecane	C_16_H_34_	1.85
6	22.53	Hentriacontane	C_31_H_64_	10.59
7	22.79	Palmitaldehyde	C_16_H_32_O	5.08
8	23.08	Tritetracontane	C_34_H_88_	2.16
9	23.86	Squalene	C_30_H_50_	3.27
10	24.52	5-Hexyl-1,4-dioxane-2-carboxylic acid	C_11_H_20_O_4_	5.18
11	25.24	Methyl 2-hydroxy-eicosanoate	C_21_H_42_O_3_	3.47
12	25.89	Cyclopentane,1,1,3-trimethyl	C_8_H_16_	2.56
13	27.38	Palmitoleic acid	C_16_H_30_O_2_	1.93
14	28.21	Vitamin E	C_29_H_50_O_2_	3.89
15	29.56	3-Methylene-1-oxa-spiro [3,6]decane	C_10_H_16_O	0.69

CSE, *Colpomenia sinuosa* extract; RT, retention time; MF, molecular formula; P.A%, peak area percentage.

### 3.3 The oral LD_50_ of CSE in rats

**FIGURE 2:**
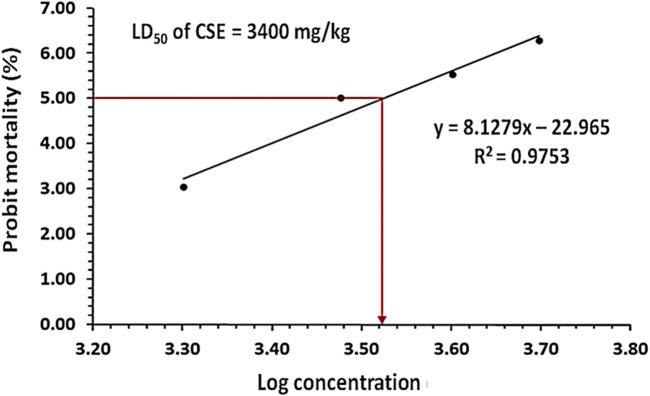
The probit analysis showed the probit mortality (%) in rats following the oral treatment with different doses of *Colpomenia sinuosa* extract (CSE) ranging from 1,000 to 5,000 mg/kg n = 4, the significant difference was at *P* < 0.05 the obtained value of the oral CSE LD_50_ was 3,400 mg/kg.

The oral LD_50_ of CSE was evaluated in rats, and probit analysis revealed that the LD_50_ was 3,400 mg/kg. Except at 3,400 mg/kg, the animals displayed no classic toxic signs including convulsions, ataxia, diarrhea, or increased diuresis (Figure 2).

### 3.4 Effect of PbAc/CSE treatment on the body weight, epididymis, and testis weights

**FIGURE 3:**
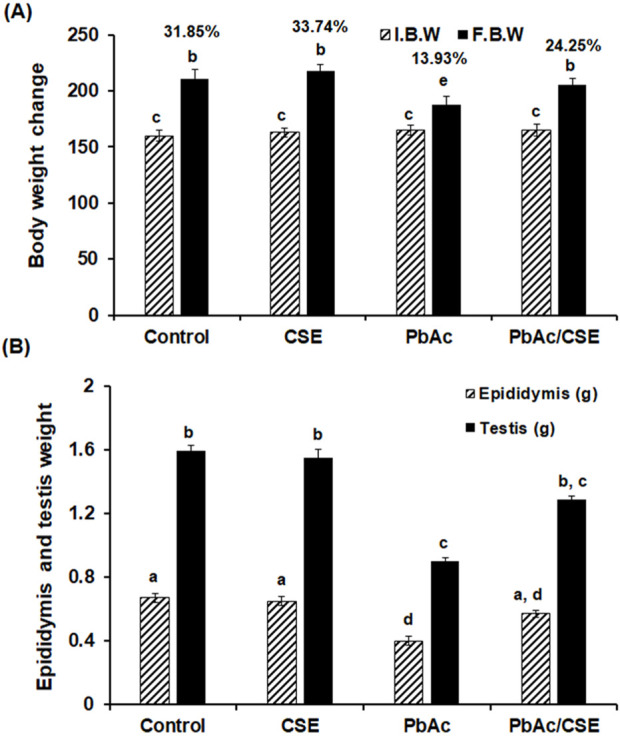
**(A)** The initial, final body weights and the percentages of the body weight change. **(B)** The epididymis and testis weights in the different groups. I.B.W: Initial body weight, F.B.W: Final body weight, CSE: *Colpomenia sinuosa* extract, PbAc: Lead acetate. Data was expressed as mean ± S.D. (n = 10). Means that do not share a letter showed significant difference (*P* < 0.05).

Administration of PbAc in rats led to a significant decrease (*P* < 0.05) in their % b. wt. change to 13.93% compared to the control and CSE received groups (31.85% and 33.74%, respectively). The significant reduction in b. wt of the animals is a common sign of systemic toxicity. This reduction in body weight typically reflects a broader systemic effect of Pb poisoning, indicating that PbAc is not only affecting the testis but also potentially impacting other organs and overall metabolic health of the animal. However, CSE helps in improving overall health, reducing systemic toxicity, and preventing weight loss. The group that was treated with PbAc/CSE showed a significant improvement (*P* < 0.05) in their % b. wt. changes (24.25%) when compared to the PbAc-exposed group alone (Figure 3A). There were significant reductions (*P* < 0.05) in the testicular and epididymal weights of the PbAc-administered group when compared to the control groups. Treatment with CSE led to a significant improvement in the testis and epididymis weights in the PbAc-intoxicated rats (Figure 3B).

### 3.5 Effect of CSE treatment on serum and testicular Pb concentrations of PbAc-exposed rats

**FIGURE 4:**
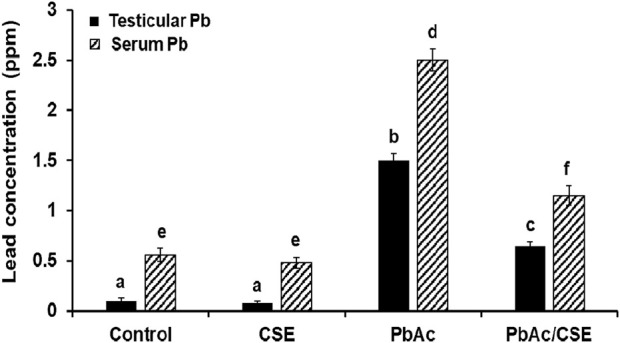
Effect of administrated PbAc/CSE on lead concentrations in the testis and serum of male rats. CSE: *Colpomenia sinuosa* extract, PbAc, Lead acetate. Data was expressed as mean ± S.D. (n = 10). Means that do not share a letter showed significant difference (*P* < 0.05).

The residual Pb in testis tissue homogenates significantly increased (*P* < 0.05) in the PbAc-exposed rats (1.5 ± 0.068 ppm) versus the control (0.1 ± 0.032 ppm) and CSE-administered (0.08 ± 0.025 ppm) groups. However, treatment with PbAc/CSE led to a significant reduction in these Pb residues to 0.65 ± 0.047 ppm. Similarly, serum Pb concentrations significantly declined (*P* < 0.05) after the treatment of PbAc-administered rats with CSE to 1.15 ± 0.096 ppm compared to the Pb-exposed rats alone (2.52 ± 0.15 ppm) (Figure 4).

### 3.6 Treatment with CSE improved spermatological parameters of PbAc-administered rats

The PbAc-administered group demonstrated the highest percentages of abnormality (24.37% ± 2.26). However, the PbAc/CSE treated rats showed a significant reduction (*P* < 0.05) in the abnormality (%) (17.13 ± 2.89) (Table 4). The sperm count of the PbAc-administered group was significantly decreased (*P* < 0.05) compared to the control groups. The treatment of PbAc-exposed group with 1/10 of CSE LD_50_ resulted in significant improvement in the sperm count compared to the PbAc-gavaged group (Table 4). The Pb intoxication in rats recorded a significant decrease (*P* < 0.05) in the motility (%) versus control groups. It was reported that sperm motility of the PbAc/CSE-treated group was not different from the control groups. Furthermore, a significant reduction in sperm viability (%) was represented in rats that were exposed to PbAc up to 32.04% ± 3.74% against the control (63.77% ± 3.49%) and CSE control (66.81% ± 3.75%) groups. However, the PbAc/CSE-treated group showed significant increase (*P* < 0.05) in the percentage of sperm viability (Table 4).

**TABLE 4:** Treatment with CSE improves spermatological alterations of lead acetate-intoxicated rats.

Groups	Abnormality (%)	Count (x 10^6^/mL)	Motility (%)	Viability (%)
Control	12.65 ± 1.76^b^	74.55 ± 4.95^a^	65.18 ± 4.79^a^	63.77 ± 3.49^b^
CSE	10.49 ± 1.95^b^	79.63 ± 3.87^a^	69.83 ± 3.97^a^	66.81 ± 3.75^b^
PbAc	24.37 ± 2.26^c^	40.27 ± 2.97 ^b^	38.79 ± 2.95^b^	32.04 ± 3.74^c^
PbAc/CSE	17.13 ± 2.89^d^	59.85 ± 3.26^c^	58.74 ± 3.85^a^	53.14 ± 4.27^b^

Data was expressed as mean ± S.D. (*n* = 10). CSE, *Colpomenia sinuosa* extract, PbAc: Lead acetate; Means that do not share a letter in each column showed significant difference (*P* < 0.05).

### 3.7 Treatment with CSE restores testicular steroidogenic proteins and male reproductive hormones in PbAc-administered rats

**FIGURE 5:**
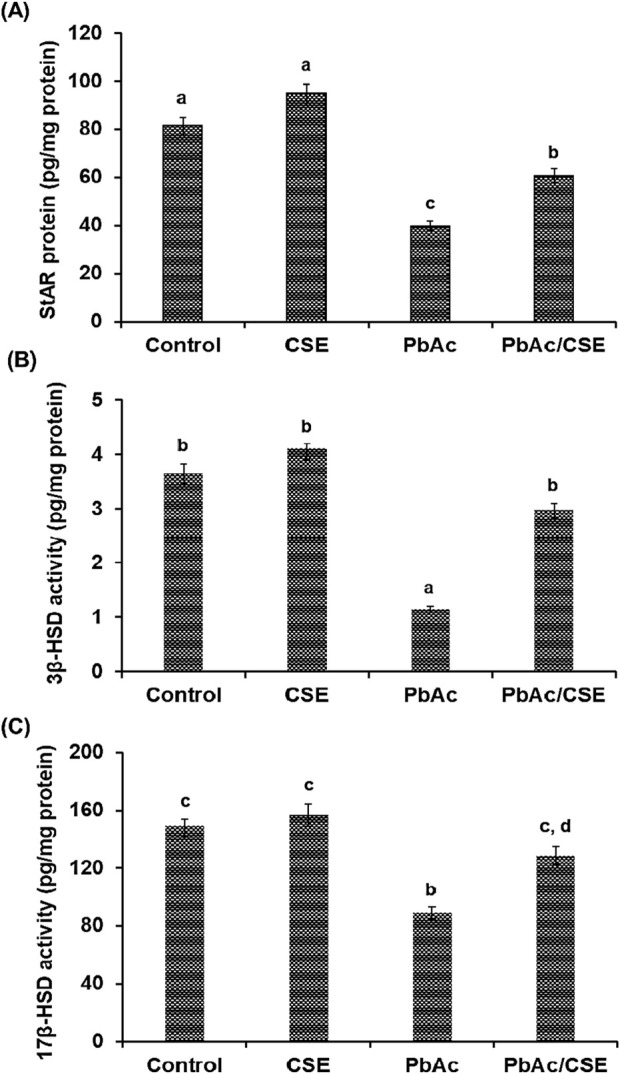
Steroidogenic acute regulatory protein (StAR) **(A)**, 3β-hydroxysteroid dehydrogenase (3β-HSD) **(B)**, and 17β-HSD **(C)** in the testicular homogenates of the different groups. CSE: *Colpomenia sinuosa* extract, PbAc: Lead acetate. Data was ex-pressed as mean ± S.D. (n = 10). Means that do not share a letter showed significant difference (*P* < 0.05).

Testicular StAR protein level was significantly decreased (*P* < 0.05) in the PbAc-treated group (39.75 ± 2.25 pg/mg protein) versus the negative control (81.65 ± 3.68 pg/mg protein) and CSE control (94.89 ± 3.57 pg/mg protein) groups; however, their levels were significantly increased (*P* < 0.05) in the Pb + CSE group (60.65 ± 2.85 pg/mg protein) compared with PbAc-gavaged group alone (Figure 5A). Furthermore, 3β-HSD and 17β-HSD activities were markedly reduced (*P* < 0.05) in testis homogenates post PbAc treatment in rats, treatment of PbAc-administered rats with CSE restored their activities close to the control groups (Figures 5B, C).

**FIGURE 6:**
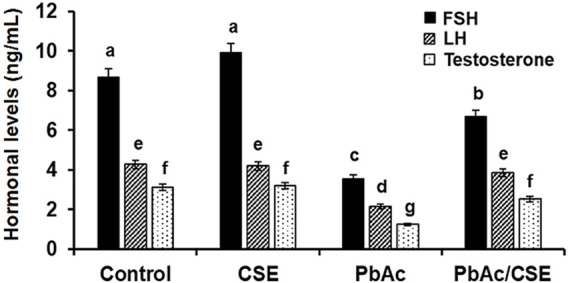
Serum levels of Follicular stimulating hormone (FSH), Luteinizing hormone (LH), and Testosterone in the different groups. CSE: *Colpomenia sinuosa* extract, PbAc: Lead acetate. Data was expressed as mean ± S.D. (n = 10). Means that do not share a letter showed significant difference (*P* < 0.01).

Treatment with 100 mg/kg PbAc orally in rats led to significant reductions (*P* < 0.01) in the FSH, LH, and testosterone hormonal levels (3.56 ± 0.12, 2.15 ± 0.10, and 1.25 ± 0.08 ng/mL, respectively) compared to the normal control (8.65 ± 0.45, 4.28 ± 0.34, and 3.12 ± 0.28 ng/mL, respectively) and CSE control (9.89 ± 0.62, 4.19 ± 0.30, and 3.20 ± 0.19 ng/mL, respectively) groups. Treatment of PbAc-exposed rats with CSE led to significant improvements (*P* < 0.01) in the LH, FSH, and testosterone serum levels (Figure 6).

### 3.8 Treatment with CSE mitigates pbAc-induced oxidative stress in rats’ testis

The levels of SOD, CAT, and SDH were significantly declined (*P* < 0.05) in the PbAc-exposed rats when compared to their control groups. However, these reductions were significantly restored in the PbAc/CSE-treated group. In contrast, the levels of testicular MDA and PC were significantly increased (*P* < 0.05) in the PbAc-administered rats by 2.6 and 3 folds, respectively (Table 5). Treatment with 1/10 of CSE LD_50_ in the PbAc-intoxicated group showed a significant increase (*P* < 0.01) in their testicular GSH, GPX, and GST protein levels compared to the PbAc-exposed rats alone. Moreover, the treatment with CSE led to a dramatic reduction (*P* < 0.01) in the testicular LDH close to the control groups compared to the PbAc-exposed rats alone (Table 5).

**TABLE 5:** Effect of CSE on testicular oxidative stress of lead acetate-treated rats.

Groups	SOD (U/mg protein)	CAT (U/mg protein)	SDH (mmol/mg protein)	MDA (nmol/g tissue)	PC (nmol/mg protein)
Control	0.95 ± 0.08^b^	4.79 ± 0.32^a^	28.69 ± 2.44^b^	23.14 ± 1.87^a^	2.63 ± 0.27^c^
CSE	1.12 ± 0.11^b^	5.18 ± 0.41^a^	31.98 ± 2.92^b^	19.65 ± 1.89^a^	2.15 ± 0.19^c^
PbAc	0.39 ± 0.05^c^	2.05 ± 0.19^b^	10.79 ± 1.23^a^	52.17 ± 3.93^e^	7.52 ± 0.61^b^
PbAc/CSE	0.67 ± 0.07^b,d^	3.48 ± 0.28^a,c^	20.75 ± 2.05^b,c^	31.58 ± 2.33^d^	3.72 ± 0.450^a^

Groups	GSH (mmol/g tissue)	GPX (U/mg protein)	GST (U/mg protein)	LDH (U/L)
Control	44.67 ± 2.85^a^	53.48 ± 2.76^e^	162.81 ± 4.21^b^	18.79 ± 0.94^a^
CSE	49.48 ± 3.49^a^	60.12 ± 3.27^e^	173.59 ± 3.96^b^	14.83 ± 0.88^a^
PbAc	21.24 ± 1.63^d^	28.19 ± 1.97^b^	126.73 ± 3.45^a^	41.90 ± 1.96^b^
PbAc/CSE	33.97 ± 2.38^c^	40.74 ± 2.83^a^	143.52 ± 3.39^b,c^	22.68 ± 1.25^a^

Data was expressed as mean ± S.D. (*n* = 10). ; SOD, superoxide dismutase; CAT, catalase; MDA, malondialdehyde; PC, protein carbonyl; SDH, sorbitol dehydrogenases; CSE, *Colpomenia sinuosa* extract, PbAc: Lead acetate; Means that do not share a letter in each column showed significant difference (*P* < 0.05).

Data was expressed as mean ± S.D. (*n* = 10). GSH, reduced glutathione; GPX, glutathione peroxidase; GST, Glutathione-S-transferase; GR, glutathione reductase; LDH, lactate dehydrogenase; CSE, *Colpomenia sinuosa* extract, PbAc, Lead acetate; Means that do not share a letter in each column showed significant difference (*P* < 0.01).

### 3.9 CSE exert anti-inflammatory properties in PbAc-testicular injured rats

The results revealed that the levels of inflammatory cytokines, including TNF-α, NF-κB, IL-6, and COX-2 were significantly increased (*P* < 0.001) in the testicular homogenates of the PbAc-injured rats compared to the negative and CSE control groups. However, the PbAc/CSE-treated group demonstrated significant restoration (*P* < 0.001) in these inflammatory biomarkers close to the control groups (Table 6).

**TABLE 6:** Effect of CSE on the testicular inflammatory biomarkers of lead acetate-administered rats.

Groups	TNF-α (Pg/ml/mg protein)	NF-κB (ng/mg tissue)	IL-6 (Pg/ml/mg protein)	COX-2 (ng/g protein)
Control	65.83 ± 2.15^c^	26.79 ± 1.57^b^	38.91 ± 1.62^a^	249.79 ± 5.93^d^
CSE	60.79 ± 1.95^c^	20.95 ± 1.21^b^	32.27 ± 2.13^a^	235.33 ± 4.62^d^
PbAc	95.68 ± 3.24^d^	59.08 ± 3.39^a^	77.55 ± 3.25^b^	336.18 ± 6.89^b^
PbAc/CSE	70.43 ± 2.75^c^	39.12 ± 2.87^c^	44.19 ± 3.02^a^	297.66 ± 5.88^c^

Data was expressed as mean ± S.D. (*n* = 10). ; TNF-α: tumor necrosis factor alfa; NF-κB: nuclear factor kappa beta; IL-6: Interleukin 6; IL-1β: Interleukin 1 beta; COX-2: Cy-clooxygenase-2; CSE: *Colpomenia sinuosa* extract, PbAc: Lead acetate; Means that do not share a letter in each column showed significant difference (*P* < 0.001).

### 3.10 Treatment with CSE targeting inflammation and apoptosis by modulating JAK2/STAT3, NLRP3/caspase-1 pathways, in PbAc-injured rats

**FIGURE 7:**
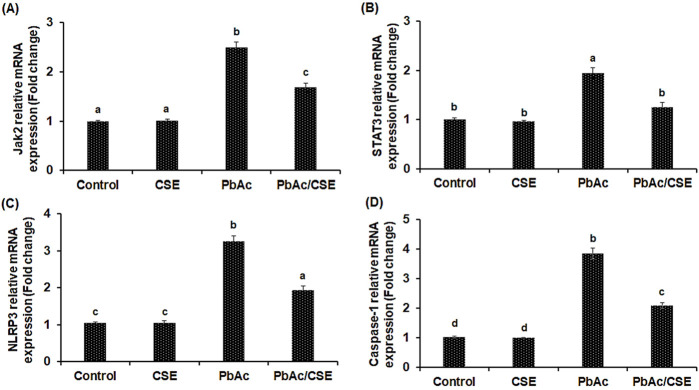
Relative mRNA expression levels of Janus kinase-2 (JAK2) **(A)**; Signal transducer and activator of transcription-3 (STAT3) **(B)**; Nucleotide-binding domain, leucine-rich-containing family, pyrin domain-containing-3 (NLRP3) **(C)**; Caspase-1 **(D)**. CSE: *Colpomenia sinuosa* extract, PbAc: Lead acetate. Data was expressed as mean ± S.D. (*n* = 10). Means that do not share a letter showed significant difference (*P* < 0.01).

In the PbAc-injured rats, the mRNA expressions of JAK2, STAT3, NLRP3, and Caspa-se-1 were significantly upregulated compared to control groups, however, PbAc/CSE treatment led to significant downregulation of these genes (*P* < 0.01). Furthermore, the PbAc-administered group showed over-expression in the pro-apoptotic gene (Bax) ac-companied by downregulation of the anti-apoptotic gene (Bcl2) when compared to control groups (*P* < 0.01), treatment of PbAc-exposed rats with CSE restored these alterations in the relative expression levels of the apoptosis-related genes (Figure 7).

**FIGURE 8:**
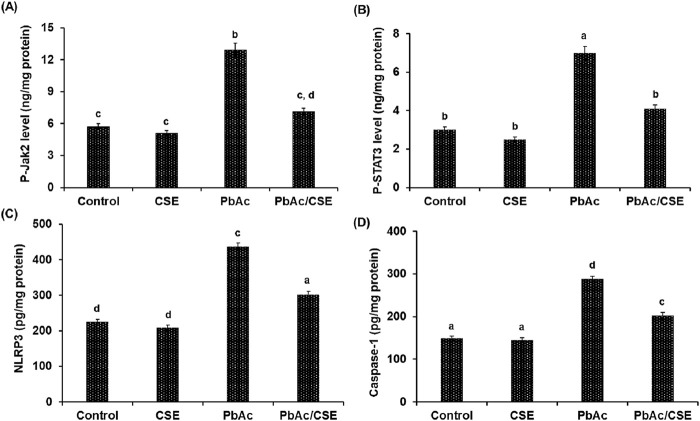
The protein levels of p-Jak2 **(A)**, p-STAT3 **(B)**, NLRP3 **(C)**, and Caspase-1 **(D)**. CSE: *Colpomenia sinuosa* extract, PbAc: Lead acetate. Data was expressed as mean ± S.D. (*n* = 10). Means that do not share a letter showed significant difference (*P* < 0.05).

Evaluation of the PbAc/CSE treatment on the amount of activated Jak2 and STAT3, determined by phosphorylation of both the tyrosine (Tyr570) and serine (Tyr705), respectively showed significant increase (*P* < 0.05) in Jak2 and STAT3 phosphorylation of the PbAc-injured group compared to the negative and CSE control groups. However, treatment with CSE resulted in significant reduction (*P* < 0.05) in p- Jak2 and p- STAT3 in the testicular tissue homogenate of PbAc-administered rats when compared to PbAc-intoxicated rats alone (Figure 8).

### 3.11 Interactions of the most bioactive compounds in CSE with JAK2, STAT3, NLRP3, and caspase-1 proteins by molecular docking

**FIGURE 9:**
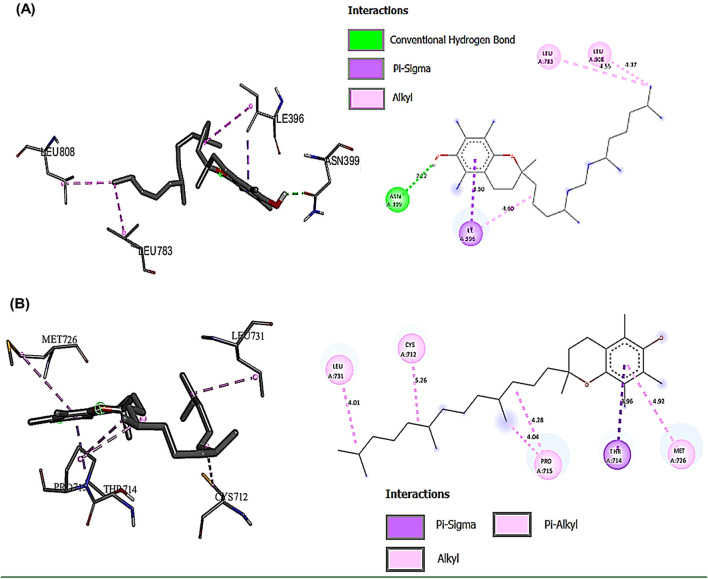
Showed the best compounds interactions with JAK2 and STAT3 proteins (2D) and (3D). **(A)** Vitamin E with JAK2 (−7.1). The conventional hydrogen bond (ASN399 -2.23A), hydrophobic interactions, Pi-Sigma (ILE396 -3.89A), and Pi-Alkyl (LEU783 -4.54 and LEU808 -5.36A). **(B)** Vitamin E with STAT3 (−6.1). The hydrophobic interactions, Pi-Sigma (THR714 -3.96A), and Pi-Alkyl (CYS712 -5.15, PRO715 -4.28A, LEU731 -4.01A, and MET726 -4.92A).

The binding interactions between various bioactive compounds in CSE and key proteins JAK2, STAT3, NLRP3, and Caspase-1 showed that among the tested compounds (Table 7). Vitamin E emerged as the most promising ligand, demonstrating remarkable binding affinities across multiple targets, particularly with JAK2 (−7.1 kcal/mol) and NLRP3 (−7.7 kcal/mol) (Table 7). In the case of JAK2, Vitamin E’s strong binding affinity (−7.1 kcal/mol) is supported by a crucial hydrogen bond with ASN399 (2.22098 Å), which appears to anchor the molecule in the binding pocket. The presence of multiple hydro-phobic interactions with ILE396, LEU783, and LEU808 suggests a binding mode that could interfere with ATP binding or alter the kinase domain’s conformation. This interaction pattern indicates potential inhibition of JAK2’s catalytic activity (Figure 9A). The STAT3-Vitamin E complex (−6.1 kcal/mol) displayed an interesting pattern of predominantly hydrophobic interactions, including contacts with CYS712, PRO715, and LEU731. The Pi-Sigma interaction with THR714 adds another layer of stability to the complex (Figure 9B).

**TABLE 7:** The binding affinity and ∆G (Kcal/mol) for JAK2, STAT3, NLRP3, and Caspase-1 proteins with active compounds.

Compound	JAK2	STAT3	NLRP3	Caspase-1
2-Hexadecanol	−4.4	−4.5	−4.8	−3.9
L-(+)-Ascorbic acid	−5.4	−5	−6	−4.1
N-propyl 11-octadecenoate	−5.1	−4.6	−5.2	−3.7
3-Methyl-2-(2-oxopropyl)furan	−5	−4.6	−4.8	−4.3
Hexadecane	−4.2	−4.4	−4.5	−3.1
Hentriacontane	−4.5	−4.1	−5.7	−4
Palmitaldehyde	−4.3	−4.1	−5.4	−3.7
Tritetracontane	−4.3	−3.1	−5.5	−3.2
Squalene	−6	−6	−7.5	−5.9
5-Hexyl-1,4-dioxane-2-carboxylic acid	−5.4	−5.3	−5.4	−4.2
Methyl 2-hydroxy-eicosanoate	−4.2	−4.8	−5.3	−4.3
Cyclopentane,1,1,3-trimethyl	−5.4	−5.3	−5.4	−4.8
Palmitoleic acid	−4.9	−4.5	−5.2	−3.8
Vitamin E	−7.1	−6.1	−7.7	−5.1
3-Methylene-1-oxa-spiro [3,6]decane	−5.5	−5.3	−5.4	−4.1

JAK2, Janus kinase-2; STAT3, Signal transducer and activator of transcription-3; NLRP3, Nucleotide-binding domain, leucine-rich-containing family, pyrin domain-containing-3.

**FIGURE 10:**
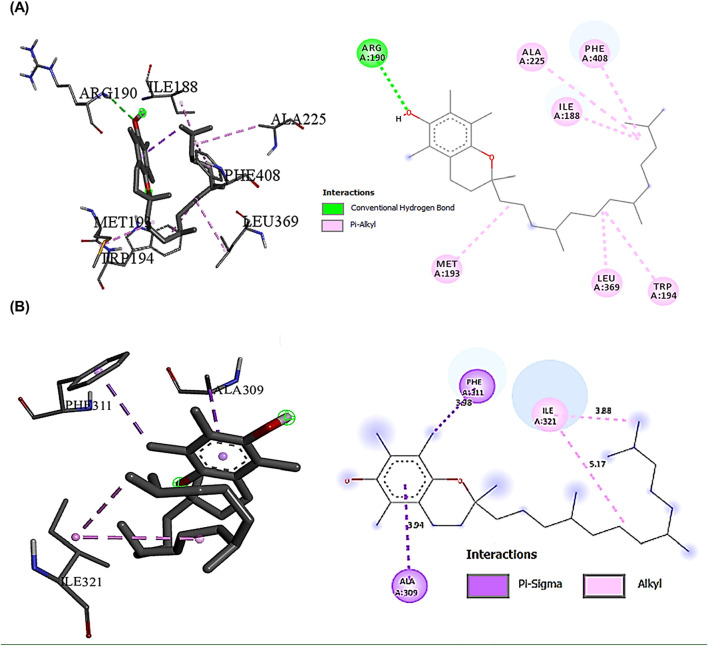
Showed the best compounds interactions with NLRP3, and Caspase-1 proteins (2D) and (3D). **(A)** Vitamin E with NLRP3 (−7.7). The conventional hydrogen bond (ARG190 -2.79A), the hydrophobic interactions, Pi-Alkyl (ILE188 -4.04A, MET193 -4.74A, ALA225 -5.31A, LEU369 -4.45A, TRP194 -4.62A, and PHE408 -5.14A). **(B)** Squalene with Caspase-1 (−5.9). The hydrophobic interactions Pi-Alkyl (ILE321-4.27A), Pi-Sigma (ALA309-3.64A) and (PHE311-3.53A).

The interaction profile of Vitamin E with NLRP3 revealed a complex network of binding interactions that may explain its potent anti-inflammatory properties. The conventional hydrogen bond formation between ARG190 and Vitamin E (2.79555 Å) likely stabilizes the binding pocket, while multiple hydrophobic interactions with residues ILE188, MET193, ALA225, and LEU369 create a stable hydrophobic cage (Figure 10A). Squalene, while showing lower overall binding affinities compared to Vitamin E, demonstrated notable specificity for Caspase-1 (−5.9 kcal/mol). The extensive network of hydro-phobic interactions with LEU267, ILE321, and ILE316 suggested a binding mode that could influence the proteolytic activity of Caspase-1 (Figure 10B).

### 3.12 CSE treatment counter histopathological changes in PbAc-testicular injured rats

**FIGURE 11:**
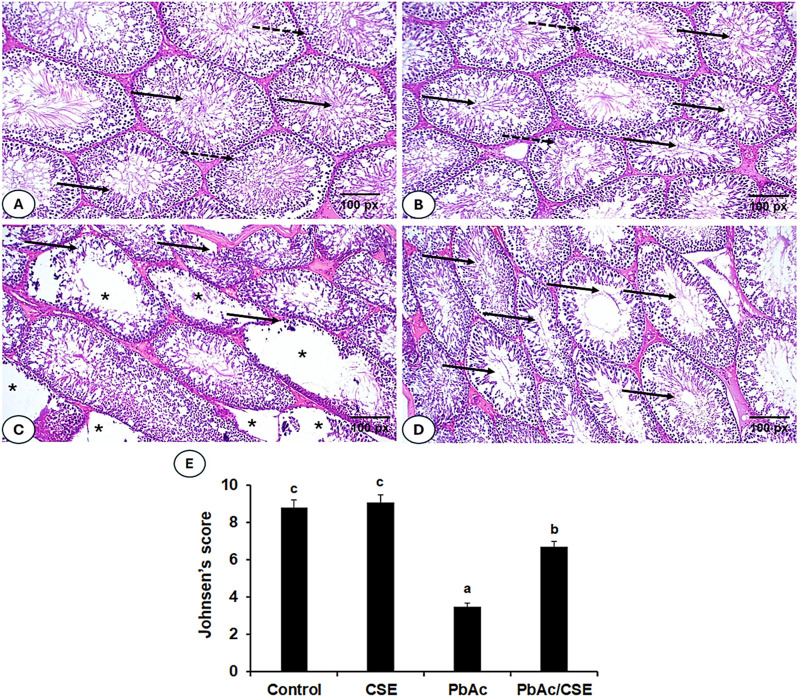
**(A–D)**. Photomicrographs of testis sections from different groups. **(A, B)** Shows testis sections from the negative and CSE control groups, indicating normal testicular architectures, normal luminal sperms (solid arrow), and spermatogonial layers (dotted arrow). **(C)** Shows a section of the PbAc-injured group, with congested testicular vessels, defective spermatogenic series in the seminiferous tubules (*), irregular seminiferous tubules (solid arrow), and degeneration of spermatogenic epithelial series. **(D)** Shows testicular sections of the PbAc/CSE-treated group with a mitigative effect of testicular histopathology, restored seminiferous tubule structures, and maintained spermatogonia cells. (H & E stain, magnification = ×200). **(E)** Effects of PbAc/CSE treatment on Johnsen testicular score (*n* = 3). CSE: *Colpomenia sinuosa* extract, PbAc: Lead acetate; Data was expressed as mean ± S.D. Means that do not share a letter showed significant difference (*P* < 0.001).

Testicular sections from the negative control and the CSE control groups exhibited normal histological architectures of active Sertoli cells, mature seminiferous tubules, and regular interstitial Leydig cells (Figures 11A, B). In contrast, sections of the PbAc-administered rats showed great testicular tissue alterations, testicular vessels congestion, irregular, damaged basement membrane, absence of spermatogenic series, disrupted seminiferous tubules, and severe degeneration of the spermatogoneal cells (Figure 11C). A significant restoration of most seminiferous tubes’ architectures, spermatogenesis process with normal-like Leydig cells was observed in testicular sections of PbAc/CSE-treated rat (Figure 11D). The Johnsen score of the testis sections from the PbAc-administered group showed a significant reduction (*P* < 0.001) compared to the negative and CSE control groups; however, the score results of the PbAc + CSE group significantly increased (*P* < 0.001) (Figure 11E).

## 4 Discussion

Toxic heavy metal poisoning is a serious environmental issue with health implications (Mitra et al., 2022). The Pb is one of the most hazardous heavy metals among the ten hazardous compounds because of its diverse applications, which continue to pose a significant threat to human health (Witkowska et al., 2021). Exposure to Pb causes inhibition of testicular functions due to oxidative stress, which in turn impairs spermatogenesis (Abu-Khudir et al., 2023). Chelating medications were used to treat the toxicity of heavy metals. However, most of these medications have unfavorable side effects. Therefore, developing safe and natural agents to alleviate poisoning is essential (El-Said et al., 2022; Hussein et al., 2024). Brown seaweed has shown potent biological activities due to its contents of biogenic molecules. *C. sinuosa*, the biomass of marine algae, was used as an adsorbent for heavy metal removal from contaminated water (Cirik et al., 2012; Al Monla et al., 2022a). There have been no studies conducted in the Mediterranean to evaluate the mechanisms of *C. sinuosa*-derived compounds in heavy metals chelation (Al Monla et al., 2020a). The purpose of this investigation for the first time was to address the mechanisms of the ameliorative effect of CSE treatment versus the male rats’ testicular dysfunctions inflicted by PbAc in rats.

The chemical components from brown alga were useful for the creation of novel medications against various ailments. In this study, CSE showed promising quantities of phytochemical constituents that agreed with prior reports that demonstrated various phyto-chemicals in *C. sinuosa* with biological potentials (Al Monla et al., 2020b; Paul, 2021). The CSE demonstrated promising *in vitro* metal chelating activity to 85%, and the DPPH scavenging capacity of CSE was 87%. These findings suggested the antioxidant potential of CSE in preventing oxidative stress-mediated pathogenesis (Al Monla et al., 2020b). Pronounced bioactive compounds were detected in CSE by using GC-MS including cocktails of 15 promising bioactive compounds determined in the present study. Among them, the major constituents were L-ascorbic acid, hentriacontane, squalene, 5-hexyl-1,4-dioxane-2-carboxylic acid, and vitamin E with the highest peak, which provides several biological activities. L-Ascorbic acid is a potent antioxidant with several applications in the pharmaceutical fields with essential physiological and metabolic activities (Caritá et al., 2020). Hentriacontane has various pharmacological properties including antimicrobial, anti-inflammatory, and antitumor potentials (Khajuria et al., 2017). Squalene is a triterpene that demonstrated potent biological and pharmacological activities, including anticancer, antioxidant, anti-inflammatory, drug carrier, and as detoxifier agent (Huang et al., 2009; Kim and Karadeniz, 2012). Vitamin E is extensively incorporated in the cell antioxidant defense system, it has been shown to be effective against numerous possible conditions and diseases, including infertility, cancer, ageing, arthritis, cataracts, and atherosclerosis (Rizvi et al., 2014).

Understanding the interactions is important for optimizing ligand structure to facilitate favorable interactions with target proteins that help in developing potent drugs (Agu et al., 2023). Molecular docking analysis showed that Vitamin E exerted the most pronounced ligand with remarkable binding affinities across multiple targets. This strong multi-target affinity suggests Vitamin E’s potential role as a master regulator in the targeted pathways. The presence of strong binding affinity and multiple hydrophobic interactions between JAK2 and Vitamin E suggested a binding mode that could interfere with ATP binding or alter the kinase domain’s conformation. This interaction pattern indicates potential inhibition of JAK2’s catalytic activity, which could lead to downstream suppression of their signaling cascades (Mengie Ayele et al., 2022). Consequently, Vitamin E form a complex with STAT3 proteins that demonstrated an interesting pattern of hydrophobic interactions. These interactions could potentially disrupt STAT3’s ability to form dimers or bind to DNA, thereby modulating its transcriptional activity and subsequent inflammatory gene expression. These findings suggest that Vitamin E may be a more promising candidate for targeting the JAK2/STAT3 pathway (Zingg, 2015; Mohd Zaffarin et al., 2020). Of particular interest is the Pi-Alkyl interaction with TRP194 and PHE408, which may contribute to the conformational changes necessary for NLRP3 inflammasome regulation. These interactions could potentially interfere with NLRP3’s ability to assemble the inflammasome complex, thereby modulating inflammatory responses. Furthermore, the extensive network of hydrophobic interactions between Squalene and Caspase-1 suggested a binding mode that could modulate Caspase-1’s proteolytic activity. This specific interaction pattern might explain the traditional anti-inflammatory and antioxidant properties attributed to squalene-containing natural products (Zhang et al., 2023).

From a toxicological perspective, the predominance of hydrophobic interactions in these complexes suggests a potentially favorable safety profile. The lack of covalent bonds and the reversible nature of these interactions indicate that these compounds might achieve therapeutic effects without causing potentially harmful permanent protein modifications. Furthermore, the observed binding energies fall within a range typically associated with therapeutic compounds (−6 to −9 kcal/mol), suggesting biological activity without excessive binding that could result in toxicity (Saini et al., 2024). The comprehensive interaction network revealed by this study pieces together a molecular puzzle of anti-inflammatory activity. The ability of Vitamin E to effectively bind multiple targets in the inflammatory cascade suggests a synergistic mechanism of action. This multi-target approach could potentially provide more robust anti-inflammatory effects compared to single-target interventions, while potentially reducing the risk of resistance development. The interaction patterns observed across all proteins indicate a preference for binding pocket regions crucial for protein function, suggesting that these compounds could modulate inflammatory responses through multiple complementary mechanisms.

In this study, the oral LD_50_ of CSE by probit analysis reported that it was 3,400 mg/kg, according to Hodge and Sterner (2005) toxicity scale, the obtained value of oral LD_50_ classified the CSE as non-toxic extract. A 1/10 of this oral CSE LD_50_ was used in our investigation for safety margin. A previous study by Atya et al. (2021) reported the hepatoprotective effect of *C. sinuosa* isolated fucoidans using doses (100 and 200 mg/kg) on paracetamol-administered rats. Administration of Pb led to a significant decrease in % b. wt., testis, and epididymis weights. These findings could be due to the toxic effects of Pb, which disrupt basal metabolism, inhibit protein production, affect steroids metabolism, and lead to testicular atrophy. These effects were consistent with previous investigations (Wani et al., 2015; Abu-Khudir et al., 2023; Mobasher et al., 2024). Treatment with PbAc/CSE led to significant improvements in the % b. wt., testis, and epididymis weights of rats, aligning with previous studies that reported similar improvements in experimental animals treated with natural products (Abd El-Hakim et al., 2018; Offor et al., 2019; Abu-Khudir et al., 2023; Mobasher et al., 2024). The CSE helps in improving overall health, reducing systemic toxicity, and preventing weight loss. Its protective effects on the testis might be secondary to its broader action in mitigating Pb-induced systemic damage. This systemic protection would enhance the ability of the testicular tissues to recover, as less overall stress would be placed on the animal’s metabolism, immune system, and other organs. In this study, elevated serum and testicular Pb concentrations were noted, indicating the accumulation of heavy Pb metals in the testicular tissues after crossing the blood-testis barrier. These levels were significantly reduced by treating PbAc-injured rats with CSE, suggesting the removal of harmful Pb effects from the rat’s tissues and circulation. This highlights the beneficial chelating properties of CSE against PbAc toxicity (Abd El-Hakim et al., 2018; Abu-Khudir et al., 2023; Mobasher et al., 2024). In the present investigation, all spermatological parameters were severely influenced by Pb treatment including, sperm motility, viability, and abnormalities (%). The morphological examination of spermatozoa indicated that head aberrations were more noteworthy than those in the tail and intermediates. This could be an indication of oxidative stress, which in turn reduced antioxidants and resulted in lipid peroxidation in cellular membranes (Pandya et al., 2012). However, treatment with CSE led to significant restoration of these spermatological parameters in PbAc-injured rats, suggesting the effects of CSE on the functional maturity of sperm. These findings were in line with previous studies investigating the impact of natural constituents on reproductive metrics of PbAc-induced testicular injury (Offor et al., 2019; Ibrahim et al., 2021; Mobasher et al., 2024). Consistently, this study reported significant decreases in the testosterone level and downregulation of activities of some steroidogenic enzymes (3β-HSD and 17β-HSD) following PbAc exposure to Pb (Figures 7, 8). This decrease could be attributed to the downregulation of StAR protein level, which is essential in the steroidogenesis process and facilitates cholesterol transfer into the Leydig cell mitochondria for the biosynthesis of testosterone. The CSE extract exerted the capability to facilitate steroidogenesis as activities of 3β-HSD and 17β-HSD and levels of StAR protein and testosterone were upregulated in comparison with PbAc-injured rats (Udefa et al., 2020; Ibrahim et al., 2021). Moreover, consistent with previous study, our data revealed that the toxic effect of Pb could be due to the direct attack of testicular Sertoli cells and an indirect effect on the hypothalamic-pituitary-testicular axis, which led to significant reduction in testosterone, FSH, and LH. These findings supported impairment in spermatogenesis and reproductive toxicity due to nutrient deficiency that led to decreased sperm viability (Offor et al., 2019; Udefa et al., 2020). Treatment with CSE showed a significant improvement in the LH and FSH secretions by gonadotrophs, which in turn restored testosterone levels through the direct action on the Leydig cells to facilitate testosterone biosynthesis. This capability of CSE to increase testosterone level could be due to the presence of potential antioxidants like hentriacontane, squalene, vitamins C, and E (Udefa et al., 2020; Ibrahim et al., 2021; Zhao et al., 2023).

The Pb is known to induce oxidative stress by releasing reactive oxygen species (ROS) from cellular membranes, including lipid peroxide, superoxide radical, and hydrogen peroxide, which led to defective scavenging activities (Zhao et al., 2023). These radical generations in spermatozoa react with polyunsaturated fatty acids in spermatozoal lipid membranes leading to lipid peroxidation, which in turn resulting in significant cellular damage and impaired semen parameters due to the binding of deleterious products with DNA and proteins (Aitken et al., 2022; Chakraborty and Roychoudhury, 2022). This study demonstrated significant elevation in the level of MDA, PC, and LDH levels, suggesting the lipid peroxidative degradation of the testicular membranes. The increase in LDH could be a compensatory mechanism to support spermatogenesis amidst Pb toxicity (Abarikwu and Farombi, 2016). On the other hand, marked depletions in the antioxidant reserves including, SOD, CAT, SDH, GSH, GPX, and GST after PbAc treatment in male rats were reported (Offor et al., 2019; Abu-Khudir et al., 2023; Mobasher et al., 2024). These decreases may result from Pb binding to the metal cofactors of these enzymes and their SH group, reducing overall function (Salama et al., 2016). The treatment with CSE led to significant restoration of the antioxidant defense system of the PbAc-testicular injured rats. This could be due to the richness of vitamin E in CSE, which is a potent antioxidant, reducing oxidative stress, and scavenging ROS, thereby protecting testicular cells from oxidative damage. These findings supported the potential antioxidant power of CSE, which may be attributed to defending the testicular tissues against the oxidative stress induced by PbAc in rats. These results were consistent with previous studies, reporting the impacts of naturally occurring compounds including algae, in attenuating the oxidative injuries induced by Pb toxicity (Ibrahim et al., 2021; Oyeyemi et al., 2022). Oxidative stress generates cellular signals’ cascades that trigger many inflammatory processes and elevated proinflammatory cytokines in the testicular tissues causing reproductive dysfunctions (Dutta et al., 2021). Inflammation caused by prostaglandin production and cyclooxygenases results in testicular hypertrophy and testosterone decline (Matzkin et al., 2021). A previous study reported that vitamin C ameliorates PbAc-induced testicular inflammatory damage via inhibiting oxidative stress mediated NF-κB signaling in mice (Zhao et al., 2023). Treating PbAc-injured rats with CSE led to significant palliative effects on testicular inflammation in rats. The anti-inflammatory and antioxidative properties of Squalene that was detected in CSE could play an important role in reducing inflammation and modulating key signaling pathways involved in testicular protection against PbAc exposure. This suggested the anti-inflammatory activities of CSE on the inflammation process promoted by Pb in the testicular tissues of male rats (Zhao et al., 2023; Tuncer et al., 2023; Mobasher et al., 2024).

Binding inflammatory cytokines to their receptors led to the activation of the JAK-STAT signaling pathway (O’Shea et al., 2013). This signaling is necessary for the testis to proliferate and consistently produce sperm. Additionally, the JAK/STAT pathway was found to be activated in the presence of reproductive toxins, which allowed germ cells to survive. Inhibiting JAK2 phosphorylation and subsequent inactivation reduced testicular injury and spermatogenesis. Therefore, JAK2 activation during testicular oxidative stress, which triggers apoptosis, is a cause of testicular dysfunction (Wu et al., 2019). This study indicated that rats exposed to PbAc exhibited increased JAK2/STAT3 in their testicular homogenates at the gene and protein expression levels associated with spermatogenic arrest and reduced expression of the Sertoli cells junctions. CSE inhibited JAK2 and STAT3 thereby maintaining spermatogenesis integrity. Furthermore, normalizing the expression of the NLRP3/Caspase-1 axis inhibited cell death. The results emphasized the regulatory role of JAK2/STAT3, NLRP3/Caspase-1 signaling in testicular tissue maintenance to resist PbAc-induced dysfunctions and male infertility (Alnajem and Al-Maghrebi, 2023; Arab et al., 2024). The mRNA expression of the anti-apoptosis gene Bcl2 was downregulated, while the pro-apoptosis Bax mRNA expression was upregulated in the PbAc-injured rats confirming the induction of apoptosis in the PbAc-subjected testis. The CSE treatment normalized the modulated mRNA expression of Bax and Bcl2 in the PbAc-administered rats. These findings were in line with previous studies which demonstrated the ameliorative effects of natural constituents against testicular damage by improving biochemical and apoptotic profile in experimental animals (Abd El-Hakim et al., 2018; Udefa et al., 2020; Ijaz et al., 2021). The current study revealed significant improvements in sperms characteristics and showed the normal histological structure of most seminiferous tubules upon treatment with PbAc/CSE. This could be attributed to the various antioxidant constituents found in CSE that counteract the negative effects of oxidative damages induced by Pb. Johnsen’s score was used as a histopathological predictor of semen quality after different treatment indications of testicular structure disruption. In this study, we applied the criteria formulated by Johnsen to assess testicular histopathology affected by PbAc/CSE treatment. The result showed that Johnsen’s score was significantly reduced in the PbAc-administered group compared to those in the control groups, increasing the possibility of infertility in male mice. This score was restored upon treatment with PbAc/CSE indicating an improvement in testicular architecture. Previous reports have demonstrated the effects of natural extracts on testicular histological damages induced by Pb, supporting our findings (Sudjarwo et al., 2017; Ijaz et al., 2021; Abu-Khudir et al., 2023; Mobasher et al., 2024). This study suggested that CSE could offer a novel therapeutic strategy for mitigating PbAc-induced testicular dysfunctions by targeting oxidative stress, inflammation, and key signaling pathways, it is still need directly applicable in clinical practice. Further research in human models, clinical trials, and safety assessments are necessary before this extract can be considered for therapeutic use in humans. Also, optimizing CSE dosing, metabolism, and biological responses in human clinical settings is crucial. To transition from animal studies to clinical practice, randomized controlled trials in human subjects are essential. This would not only confirm the safety and efficacy of the extract in treating testicular dysfunction but also provide insights into possible side effects, interactions with other medications, and optimal therapeutic regimens.

## 5 Conclusion

**FIGURE 12:**
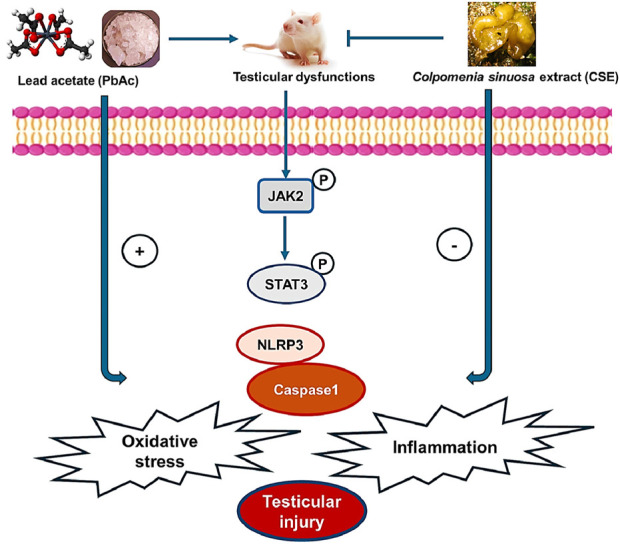
Diagram shows the effect of *Colpomenia sinuosa* extract (CSE) against lead acetate-induced testicular injury by inhibiting oxidative stress, inflammation, and modulating the JAK2/STAT3 and NLRP3/caspase1 pathways in male rats.

The JAK2/STAT3, and NLRP3/Caspase-1 pathways were implicated in Pb-induced impaired spermatogenesis and reproductive dysfunctions in male rats. Treatment with CSE significantly restored reproductive functions in PbAc-injured rats by improving testicular steroidogenic proteins, reproductive hormones, biochemical parameters, and histopathological findings. Interestingly, CSE inhibits oxidative stress, inflammation, and modulates the JAK2/STAT3, and NLRP3/Caspase-1 signaling pathways in male rats (Figure 12). However, further preclinical and clinical studies are needed to investigate the ameliorative effects of CSE against heavy metal toxicity and its underlying mechanisms. The current results serve as an encouraging starting point, but there were limitations of this study, much more work is needed before this extract can be considered a viable therapeutic option in the clinical setting to validate its pharmacokinetic properties, optimizing CSE dosing, metabolism, and biological responses, and ensure its therapeutic potential.

## Data Availability

The original contributions presented in the study are included in the article/supplementary material, further inquiries can be directed to the corresponding author.
